# DEMENTIA KNOWLEDGE, ATTITUDES, AND BELIEFS: A 3-YEAR UPDATE

**DOI:** 10.1093/geroni/igz038.1695

**Published:** 2019-11-08

**Authors:** Pedro M Santos, Constança Paúl, Catarina Alvarez, Ana Margarida Cavaleiro, Tatiana Nunes, Silvana Paules

**Affiliations:** 1 CINTESIS – Center for Health Technology and Services Research, Oporto, Portugal; 2 Alzheimer Portugal, Lisbon, Portugal

## Abstract

Objective: To characterise and analyse beliefs, knowledge, and attitudes regarding dementia three years after the first Portuguese survey (2015), in order to inform mental health research and policy-making, and establish the baseline to assess “Dementia Friends” campaign impact. Method: An advisory group, including academics and representatives of Alzheimer Portugal and Alzheimer Society UK, drafted the survey based on previous international research and the first national survey. The survey was uploaded to the Directorate-General of Health website and disseminated through relevant stakeholders and social networks. The survey was cascaded by inviting recipients to further share it. Data were collected during July 2018. Results: 1716 individuals completed the survey: median age 43y, 83.4% female, 25.1% professionals working in the field of dementia. 31.7% of respondents know someone close with dementia, and 42.5% have/had relatives with dementia. 14.7% consider people with dementia must leave/stop to attend ceremonies and social events, but this percentage increased to over one-third (37.3%) when asked about the opinion of most people living in their community. Regarding the key messages of “Dementia Friends” campaign, results reveal that 25.6% of respondents consider dementia is a natural part of ageing, 21.3% that it is not caused by diseases of the brain and 17.4% that it is just about losing memory. Conclusions: The second survey results reinforce the need for initiatives to change people’s perceptions of dementia, that build up on evidence-based decision-making and international learning and cooperation as part of “Dementia Friends” global movement.

